# The phosphorylated pathway of serine biosynthesis is crucial for indolic glucosinolate biosynthesis and plant growth promotion conferred by the root endophyte *Colletotrichum tofieldiae*

**DOI:** 10.1007/s11103-021-01181-5

**Published:** 2021-08-23

**Authors:** Sandra E. Zimmermann, Samira Blau, Henning Frerigmann, Stephan Krueger

**Affiliations:** 1 Institute for Plant Sciences, Biocenter University of Cologne, Zülpicher Straße 47b, 50674 Cologne, Germany; 2 Max Planck Institute for Plant Breeding Research, Carl-von-Linne-Weg 10, 50829 Cologne, Germany

**Keywords:** Serine biosynthesis, Indolic glucosinolate Biosynthesis, Plant growth promotion

## Abstract

**Key message:**

Phosphoglycerate Dehydrogenase 1 of the phosphorylated pathway of serine biosynthesis, active in heterotrophic plastids, is required for the synthesis of serine to enable plant growth at high rates of indolic glucosinolate biosynthesis.

**Abstract:**

Plants have evolved effective strategies to defend against various types of pathogens. The synthesis of a multitude of specialized metabolites represents one effective approach to keep plant attackers in check. The synthesis of those defense compounds is cost intensive and requires extensive interaction with primary metabolism. However, how primary metabolism is adjusted to fulfill the requirements of specialized metabolism is still not completely resolved. Here, we studied the role of the phosphorylated pathway of serine biosynthesis (PPSB) for the synthesis of glucosinolates, the main class of defensive compounds in the model plant *Arabidopsis thaliana*. We show that major genes of the PPSB are co-expressed with genes required for the synthesis of tryptophan, the unique precursor for the formation of indolic glucosinolates (IG). Transcriptional and metabolic characterization of loss-of-function and dominant mutants of *ALTERED TRYPTOPHAN1-*like transcription factors revealed demand driven activation of PPSB genes by major regulators of IG biosynthesis. Trans-activation of PPSB promoters by ATR1/MYB34 transcription factor in cultured root cells confirmed this finding. The content of IGs were significantly reduced in plants compromised in the PPSB and these plants showed higher sensitivity against treatment with 5-methyl-tryptophan, a characteristic behavior of mutants impaired in IG biosynthesis. We further found that serine produced by the PPSB is required to enable plant growth under conditions of high demand for IG. In addition, PPSB-deficient plants lack the growth promoting effect resulting from interaction with the beneficial root-colonizing fungus *Colletotrichum tofieldiae*.

**Supplementary Information:**

The online version contains supplementary material available at 10.1007/s11103-021-01181-5.

## Introduction

Plants produce thousands of different chemical structures classified as specialized metabolites, which are thought to function in facilitating the ecological interaction to optimize plant fitness (Züst and Agrawal 2017). Many of the specialized metabolites are produced in plants to repel specific attackers, but the metabolic cost for the synthesis, modification, transport, maintenance and storage of these compounds is considerable (Gershenzon 1994). Mathematic modeling revealed that maintaining the main defensive trait of plants of the Brassicaceae family, the synthesis of glucosinolates, increases the photosynthetic requirements of the plant by a minimum of 15% (Bekaert et al. 2012). Thus, the biosynthesis of glucosinolates consumes a meaningful percentage of the energy available to the growing plant and requires the development of mechanisms to mitigate the trade-off between growth and the production of defensive compounds.

Glucosinolates are nitrogen and sulfur-rich defense metabolites produced to protect plants against herbivore attack or infection with pathogenic microbes (Halkier and Gershenzon 2006; Hopkins et al. 2009; Pastorczyk and Bednarek 2016). They can be classified into three major groups: aliphatic, indolic and benzenic, depending on their respective amino acid precursor methionine, tryptophan and tyrosine/phenylalanine (Halkier and Gershenzon 2006). Intact glucosinolates are stable and show only little biological activity, while their hydrolysis by myrosinases (β-thioglucsoidases) results in the formation of a vast number of very reactive breakdown products (Wittstock and Burow 2010). Two possible pathways of glucosinolate breakdown have been described (Wittstock et al. 2016). In the “classical” pathway glucosinolates are stored in specialized cells (S-cells) and thus separated from the myrosinase, which is localized in so called myrosine cells. This spatial separation of substrate and enzyme is required to prevent premature glucosinolate breakdown, which occurs only after disruption of the plant tissue by wounding or herbivore attack (Wittstock et al. 2016). In “atypical” glucosinolate breakdown only indolic, but not aliphatic glucosinolates (AG) are hydrolyzed. The degradation of indolic glucosinolates (IG) is catalyzed by the atypical myrosinase PEN2 and occurs within intact cells (Bednarek et al. 2009). This pathway is part of the plant innate immune response and is required to control the entry of filamentous pathogens into the plant (Pastorczyk and Bednarek 2016). Furthermore, recent findings indicate that IG metabolism links plant innate immunity with phosphate nutrition in Arabidopsis plants (Hiruma et al. 2016). While the mineral nutrition of most plant species is supported by interaction with mycorrhizal fungi, around 30% of the plant species, including the Brassicaceae family, are unable to perform this symbiotic interaction (Cosme et al. 2018). However, experimental evidence indicates that other microbes bridge this gap, which has been impressively shown for the beneficial interaction of Arabidopsis plants with the growth promoting endophytic fungus *Colletotrichum tofieldiae* (*C.t.*) (Hiruma et al. 2016). The interaction with *C.t.* enables Arabidopsis plants to improve their growth and phosphate acquisition under phosphate limiting conditions, but requires a functional IG defense pathway (Bednarek et al. 2009; Hiruma et al. 2016). Mutants deficient in major components of this pathway are no longer able to establish a beneficial interaction with *C.t.* and the fungus behaves like a pathogen in these plants.

IGs are produced from tryptophan and the first reaction, the conversion of tryptophan into indole-3-acetaldoxime (IAOx), is catalyzed by CYP79B2 and CYP79B3, two redundant cytochrome P450 monooxygenase enzymes (Zhao et al. 2002). While single *cyp79b2* or *cyp79b3* mutations have no major impact on IG content, the respective double mutant is free of IGs (Zhao et al. 2002; Glawischnig et al. 2004). By multiple catalytic reactions IAOx is converted to the first IG, indole-3-ylmethyl glucosinolate (I3M), which can be further modified by hydroxylation and methylation (Pfalz et al. 2011). Hydroxylation of I3M yields in either 4-hydroxy-indole-3-ylmethyl glucosinolate (4HO-I3M) or 1-hydroxy-indole-3-ylmethyl glucosinolate (1HO-I3M), while methylation yields in 4-methoxy-indole-3-ylmethyl glucosinolate (4MO-I3M) or 1-methoxy-indole-3-ylmethyl glucosinolate (1MO-I3M) (Pfalz et al. 2009,2011).

Previous investigations discovered that IG biosynthesis is regulated by a clade of MYB transcription factors, namely MYB51/HIG1 (HIGH INDOLIC GLUCOSINOLATE 1), MYB122/HIG2 (HIGH INDOLIC GLUCOSINOLATE 2), and MYB34/ATR1 (ALTERED TRYPTOPHAN REGULATION 1) (Celenza et al. 2005; Gigolashvili et al. 2007b; Malitsky et al. 2008; Frerigmann and Gigolashvili 2014). While either their overexpression or dominant mutation increases the expression of all genes involved in IG biosynthesis and results in elevated IG levels, their loss-of-function mutation leads to a down-regulation of IG genes and reduced levels of IGs (Celenza et al. 2005; Gigolashvili et al. 2007b; Malitsky et al. 2008; Frerigmann and Gigolashvili 2014). Intense characterization of MYB34 and MYB51 further revealed their function in regulating the expression of genes of the shikimate and sulfur assimilation pathway, indicating that both transcription factors are responsible for adjusting the activity of primary metabolism to the requirements of IG biosynthesis (Malitsky et al. 2008; Yatusevich et al. 2010). Importantly, identification of tryptophan biosynthetic genes showed that the expression of these genes is induced by environmental stimuli, such as wounding or pathogen infection, and strongly correlated with the expression of genes of the IG pathway (Niyogi and Fink 1992; Niyogi et al. 1993; Zhao et al. 1998). Hence, under conditions of high demand the biosynthesis of tryptophan is boosted beyond the levels necessary for housekeeping functions, such as protein biosynthesis, to enable the synthesis of tryptophan-derived defense metabolites.

The biosynthesis of tryptophan includes six enzymatic reactions and starts with the transfer of the amino group from glutamine to chorismate, the terminal metabolite of the shikimate pathway. This reaction generates anthranilate and pyruvate and is catalyzed by the anthranilate synthase (Niyogi et al. 1993; Poulsen et al. 1993). Via multiple reactions anthranilate is further converted to indole-3-glycerol, the substrate of the tryptophan synthase complex, which consists of an alpha (TSa) and beta (TSb) subunit. The TSa cleaves indole-3-glycerol into indole and glyceraldehyde-3-phosphate, while TSb catalyzes the condensation of indole with serine to form tryptophan (Miles 2001; Weber-Ban et al. 2001). The last reaction indicates that the formation of tryptophan requires an equimolar concentration of serine. Consequently, a secured supply of serine must to be guaranteed under conditions of high tryptophan demand. In plants serine is produced by three pathways; photorespiration (PR), the phosphorylated pathway of serine biosynthesis (PPSB) and the postulated glycerate pathway (Ros et al. 2014), but it is unknown which pathway initially provides serine for tryptophan biosynthesis.

In this study, we set out to identify the mechanism which enables the plant to provide sufficient serine for the formation of tryptophan when the demand for the synthesis of IGs is high. Evidence for at least two distinct serine pools for the formation of tryptophan were previously described (Glawischnig et al. 2000; Benstein et al. 2013). Here, we show that the PPSB is co-activated with tryptophan biosynthesis and this regulation is mediated by the MYB34 transcription factor. In addition, we found that PPSB-derived serine is critical for the trade-off between plant growth and defense.

## Results

### Genes of the PPSB revealed high expression upon biotic stress and were co-regulated with tryptophan biosynthesis

**Fig. 1:**
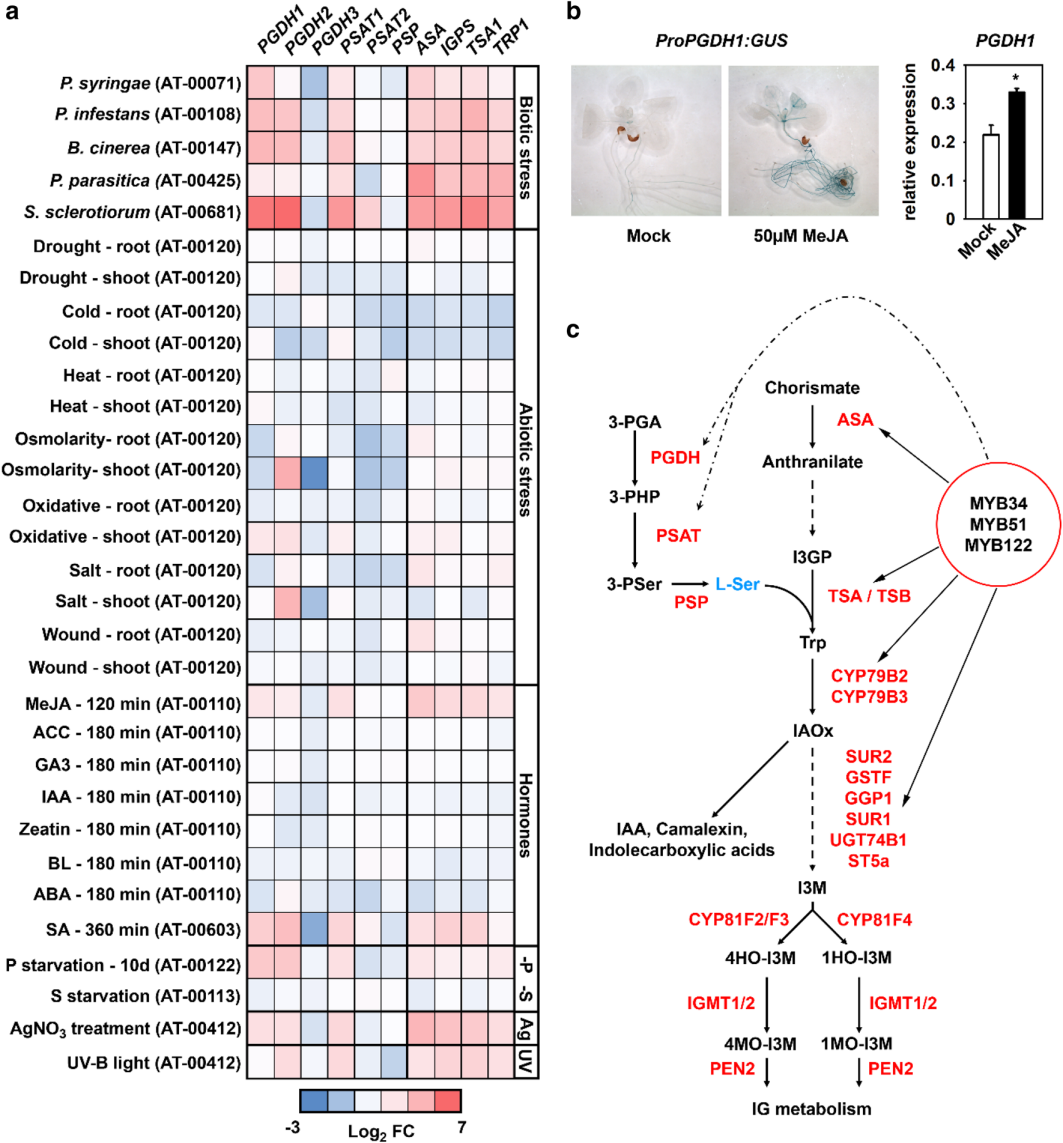
Expression analysis indicated co-regulation of PPSB and tryptophan biosynthetic genes. **a** Overview on the expression pattern of PPSB and tryptophan biosynthetic genes extracted from publicly available data sources (https://genevestigator.com). Genevestigator ID for the respective experiments are shown in brackets. **b** Histochemical staining of transgenic plants harboring the *PGDH1* promoter ß-galactosidase fusion construct after Mock and methyl jasmonate treatment (MeJA). Bar chart shows *PGDH1* expression in seedlings of wild type plants after Mock and MeJA (50 µM) treatment. Data presented are means ± SE of n = 5. Asterisks indicates significantly different values between Mock and MeJA treatment by the Student’s t test (*P < 0.05). **c** Schematic representation of the biosynthesis of serine, tryptophan, and their relation to indolic glucosinolates, indole-3-acetic acid (IAA), camalexin, and indolecarboxylic acids. The known *Arabidopsis thaliana* metabolites and proteins involved in these steps are indicated. Metabolites: *3-PGA* 3-phosphoglycerate, *3-PHP* 3-phosphohydroxypyruvate, *3-PSer* 3-phosphoserine, *I3GP* Indole-3-glycerol phosphate, *IAOx* indole-3-ylacetaldoxime, *I3M* indole-3-ylmethyl-glucosinolate, *4HO-I3M* 4-hydroxy-indole-3-ylmethyl-glucosinolate, *4MO-I3M* 4-methoxy-indole-3-ylmethyl-glucosinolate, *1HO-I3M* 1-hydroxy-indole-3-ylmethyl-glucosinolate, *1MO-I3M* 1-methoxy-indole-3-ylmethyl-glucosinolate. Multiple enzymatic steps are indicated with interrupted arrows. Proteins. *MYB34/MYB51/MYB122* ATR1-like transcription factors 34, 51 and 122, *PGDH* 3-phosphoglycerate dehydrogenase, *PSAT* 3-phosphoserine aminotransferase, *PSP* 3-phosphoserine phosphatase, *ASA* anthranilate synthase, *TSA* tryptophan synthase alpha subunit, *TSB* tryptophan synthase beta subunit, *CYP79B2/B3* cytochrome P450 monooxygenase isoform B2 and B3, *SUR2 (CYP83B1)* cytochrome P450 monooxygenase, *GSTF* glutathione S-transferase, *SUR1* C-S lyase, *UGT74B1* UDP-glucose thiohydroximate S-glucosyltransferase, *ST5a* sulfotransferase, *CYP81F2/F3/F4* cytochrome P450 monooxygenase isoform F2, F3 and F4, *IGMT1/2* glucosinolate *O*-methyltransferases isoform 1 and 2, *PEN2* beta glucosidase/myrosinase

The analysis of publicly available expression data revealed that the genes of the PPSB, namely *PGDH1*, *PGDH2* and *PSAT1* are strongly upregulated after infection of plants with pathogenic bacteria and fungi (Fig. 1a). The infection of plants with the gram-negative bacteria *Pseudomonas syringae*, the oomycetes *Phytophthora infestans* and *Phytophthora parasitica* and the necrotrophic ascomycetes *Botrytis cinerea* and *Sclerotinia sclerotiorum* elevated the expression of *PGDH1, PGDH2* and *PSAT1* of around two to seven-fold.

While biotic stress had a strong influence on the expression of these genes, no substantial changes were observed under various abiotic stresses, except for *PGDH2*, which was upregulated under osmotic and salt stress conditions (Fig. 1a).

In addition, when analyzing the phytohormone dataset within the Genevestigator Database, we found no strong influence of most phytohormone treatments on the expression of PPSB genes (Fig. 1a). Only the incubation of plants with methyl-jasmonate (MeJA) and salicylic acid (SA), two phytohormones involved in plant defense (Wasternack and Hause 2013; Ding and Ding 2020), elevated the expression of *PGDH1*, *PGDH2* and *PSAT1*. This finding was validated for *PGDH1* expression upon MeJA treatment by RT-qPCR analysis and histological staining of MeJA treated *ProPGDH1:GUS* lines (Fig. 1b).

Apart from biotic stress, MeJA and SA treatment, the expression of *PGDH1*, *PGDH2* and *PSAT1* was also significantly enhanced in plants grown under low phosphate concentrations and after treatment with AgNO_3_ or UV-B radiation (Fig. 1a).

Interestingly, the expression pattern of *PGDH1*, *PGDH2* and *PSAT1* was very similar to the expression of tryptophan biosynthetic genes, such as *ASA* (anthranilate synthase), *IGPS* (indole-3-glycerol phosphate synthase), *TSA1* (tryptophan synthase alpha subunit) and *TRP1* (phosphoribosylanthranilate transferase). The expression of these genes was also elevated in plants after pathogen infection, treatment with MeJA, SA, AgNO_3_ or UV-B and under phosphate starvation conditions (Fig. 1a). Accordingly, *PGDH1*, *PGDH2* and *PSAT1* appear to be in the same gene correlation network as major genes of the tryptophan biosynthesis pathway (Fig. S1).

The comprehensive analysis of the expression of PPSB genes revealed a strong correlation of *PGDH1*, *PGDH2* and *PSAT1* expression, while *PGDH3*, *PSAT2* and *PSP* are differentially expressed (Fig. 1a). The expression of *PGDH1*, *PGDH2* and *PSAT1* was high in roots and heterotrophic cells of the leaves, whereas *PGDH3* was specifically expressed in mesophyll cells and *PSAT2* was expressed only at low levels (Benstein et al. 2013; Toujani et al. 2013; Wulfert and Krueger 2018). PSP is not the rate limiting enzyme of the PPSB and the encoding gene is ubiquitously expressed in the plant (Benstein et al. 2013; Cascales-Minana et al. 2013). Thus, only the major genes of the heterotrophic branch of the PPSB were co-regulated with tryptophan biosynthesis and involved in the response of the plant to the above-mentioned stresses.

### Genes of the PPSB are regulated by ATR1/MYB34 transcription factor, a key homeostatic regulator of tryptophan metabolism

**Fig. 2:**
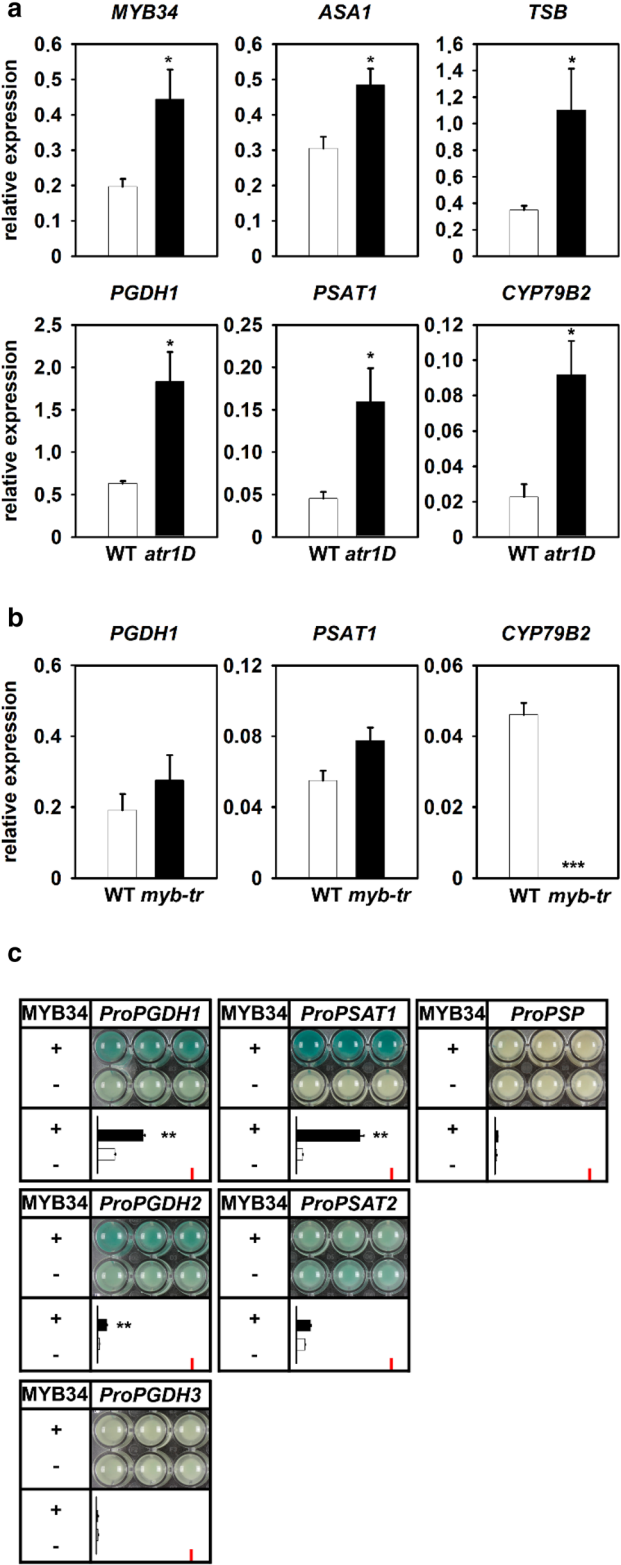
PPSB genes are regulated by *ATR1-like* transcription factors. **a** Expression of *MYB34*, *ASA*, *TSB*, *PGDH1*, *PSAT1* and *CYP79B2* in seedlings of *atr1D* dominant mutant. **b** Expression of *PGDH1*, *PSAT1* and *CYP79B2* in *myb-tr* (*myb34 myb51 myb122*) triple mutant. Data presented in (**a**) and (**b**) are means ± SE of n = 5. **c** Trans-activation of *PGDH1*, *PGDH2*, *PGDH3*, *PSAT1*, *PSAT2* and *PSP* promoter β-glucuronidase (GUS) fusions is shown. Fusion constructs were co-expressed with Pro35S:MYB34 (+) or Pro35S:[empty vector] (−) in cultured Arabidopsis root cells and the GUS activity was determined by staining (upper panel) or measuring the 4-methylumbelliferyl-β-d-glucuronidase activity in nmol (4-MU) min/mg protein (lower panel). Red bar indicates maximum of the y-axis (7 nmol (4-MU) min/mg protein). Data presented are means ± SE of n = 3 (staining) and n = 6 (4-methylumbelliferyl-β-d-glucuronidase activity). Asterisks indicate significantly different values by the Student’s t test (*P < 0.05; **P < 0.01)

To gather more information about the intimate association of PPSB and tryptophan metabolism we investigated the regulation of PPSB genes by ATR1/MYB34 transcription factor (Fig. 2a). Therefore, we analyzed the expression of *PGDH1* and *PSAT1* in the *atr1D* dominant mutant (Bender and Fink 1998; Celenza et al. 2005). The enhanced stability of the *MYB34* mRNA in the *atr1D* mutant results in elevated content of *MYB34* transcripts (Fig. 2a). Consequently, the transcript levels of the MYB34 target genes, *ASA1*, *TSB1* and *CYP79B2* were also significantly higher in the *atr1D* mutant compared to control plants (Fig. 2a). The analysis of *PGDH1* and *PSAT1* transcripts revealed substantially elevated levels in *atr1D*, indicating that also PPSB genes are regulated by the ATR1/MYB34 transcription factor (Fig. 2a).

In contrast to the elevated transcript level of *CYP79B2* gene in the dominant *atr1D* mutant (Fig. 2a), its expression was strongly reduced in the *myb-tr* (*myb34 myb51 myb122*) triple mutant (Fig. 2b). However, the expression of *PGDH1* and *PSAT1* was not significantly altered in the *myb-tr* mutant (Fig. 2b), indicating that the ATR1-like transcription factors were not the sole transcriptional regulators of PPSB gene expression.

As mentioned before *PGDH1* and *PSAT1* expression was substantially up-regulated in the *atr1D* mutant (Fig. 1a). For a detailed analysis of PPSB gene regulation by ATR1/MYB34 we performed a trans-activation assay using the Arabidopsis root cell culture system (Berger et al. 2007). Staining and fluorometric quantification of β-glucuronidase (GUS) activity showed a significant activation of *PGDH1*, *PGDH2* and *PSAT1* promoters by ATR1/MYB34, while the activity of *PGDH3*, *PSAT2* and *PSP* promoters remained unchanged (Fig. 2c).

In summary, the elevated expression of *PGDH1* and *PSAT1* in the *atr1D* mutant and the trans-activation of *PGDH1*, *PGDH2* and *PSAT1* promoters by ATR1/MYB34 indicate co-regulation of PPSB with tryptophan-dependent IG biosynthesis.

### Over-expression of ATR1/MYB34 transcription factor alters amino acid composition in Arabidopsis plants

**Fig. 3:**
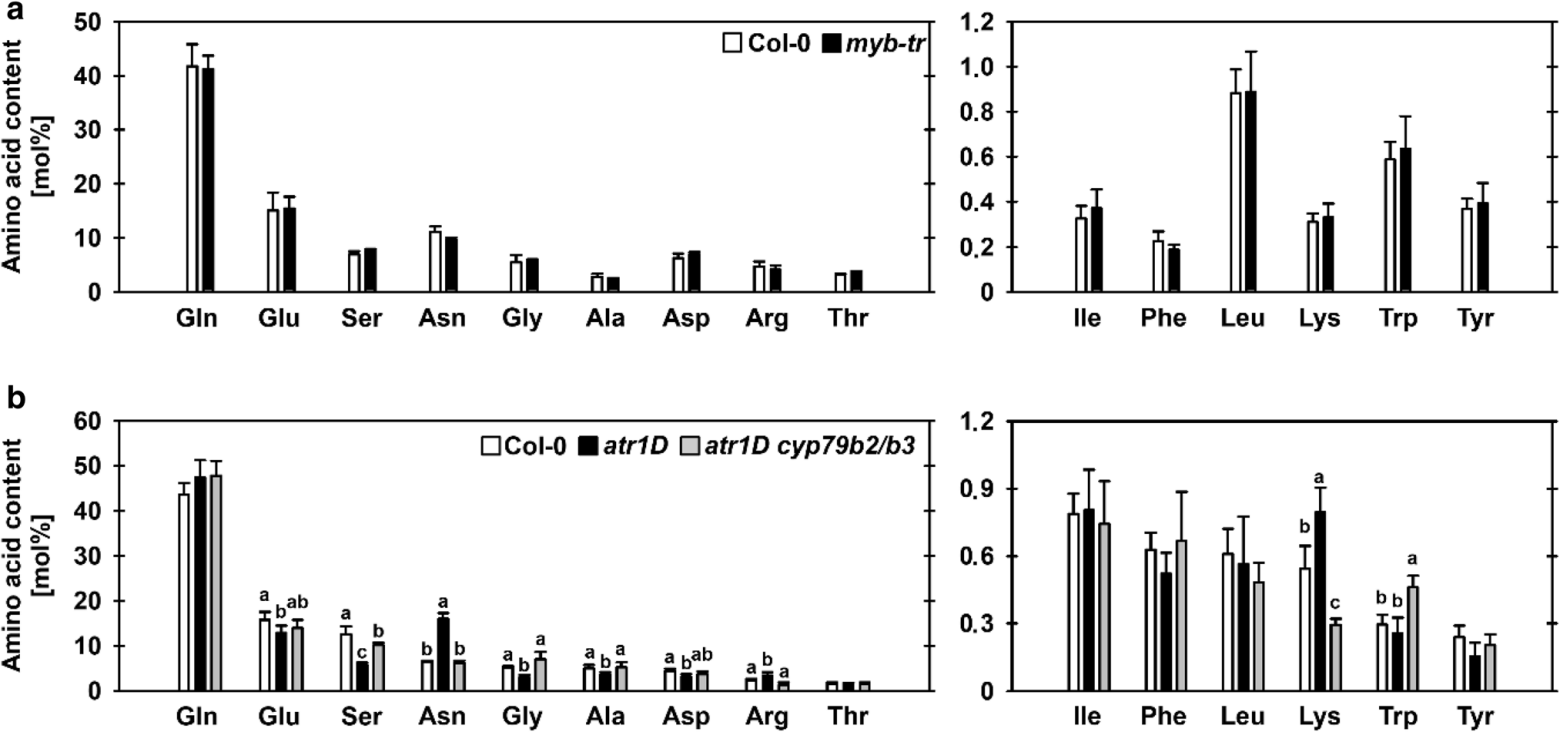
Elevated indolic glucosinolate biosynthesis alters amino acid contents in plants. The mol percentage of amino acids in seedlings of **a** *myb-tr* (*myb34 myb51 myb122*) triple mutants and of **b** *atr1D* single and *atr1D cyp79b2/b3* triple mutants is shown. Data presented are means ± SD of n = 5. Letters indicate significantly different values between control (Col-0), *atr1D* single and *atr1D cyp79b2/b3* triple mutant plants by the Student’s t test (*P* < 0.05)

Further information about the influence of the ATR1-like transcription factors on the amino acid biosynthesis were obtained by analyzing the amino acid content in *myb-tr* triple mutants (Fig. 3a) and *atr1D* mutants (Fig. 3b). The analysis of *myb-tr* triple mutants revealed no substantial changes in the amino acid content (Fig. 3a). Hence, the three MYB transcription factors were not necessarily required for maintaining amino acid biosynthesis in Arabidopsis plants, supporting its predominant role in plant specialized metabolism (Frerigmann and Gigolashvili 2014).

In contrast to the loss of MYB34/MYB51/MYB122 function, constitutive overexpression of *ATR1/MYB34* in the dominant *atr1D* mutant lead to significant changes in the level of amino acids (Fig. 3b). The levels of asparagine, arginine and lysine increased in *atr1D* mutants, while the levels of glutamate, serine, glycine, alanine and aspartate decreased. Thus, overexpression of *ATR1/MYB34* substantially alters amino acid composition in plants. However, crossing the *atr1D* mutant with the *cyp79b2/b3* (*cyp79b2 cyp79b3*) double mutant (Celenza et al. 2005) reverted almost all changes in amino acid content (Fig. 3b), indicating that the enhanced metabolic flux into the IG biosynthesis in the *atr1D* mutant was responsible for the amino acid phenotype. This hypothesis was supported by higher tryptophan content in *atr1D cyp79b2/b3* triple mutants (Fig. 3b), which most likely originates from elevated synthesis in combination with impaired turnover into IGs.

Taken together, the *ATR1*-like clade of transcription factors are not necessarily required to maintain amino acid biosynthesis in Arabidopsis. However, the high demand of amino acid-bound nitrogen for the elevated IG biosynthesis in *atr1D* mutants causes substantial alterations in amino acid biosynthetic pathways.

### A functional PPSB is required for the synthesis of indolic glucosinolates

**Fig. 4:**
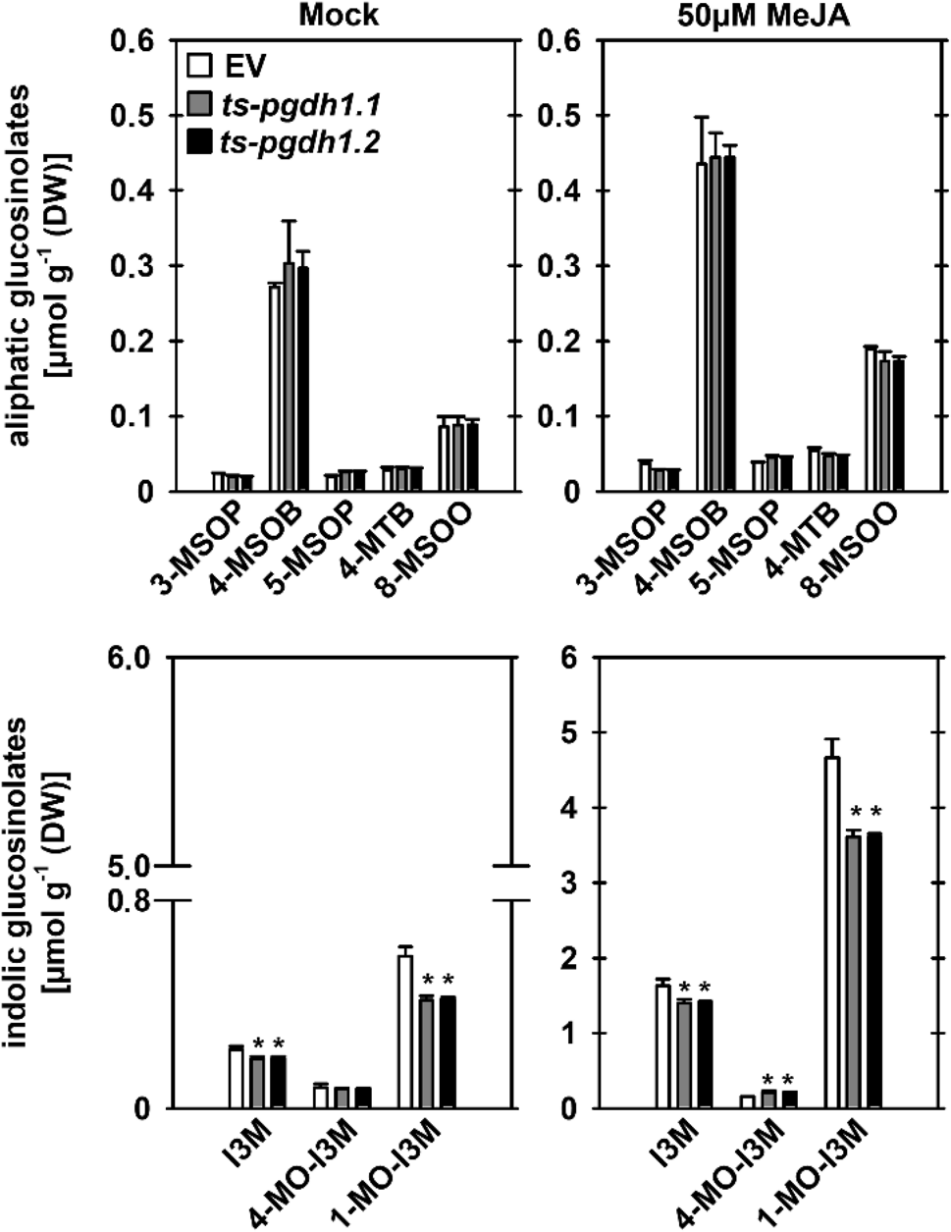
*PGDH1*-silenced lines contain lower amounts of indolic glucosinolates. The concentration of aliphatic (3-MSOP, 4-MSOB, 5-MSOP, 4-MTB, 8-MSOO) and indolic glucosinolates (I3M, 4-MO-I3M, 1-MO-I3M) was determined in seedlings of empty vector control plants (EV) and *PGDH1*-silenced lines (*ts-pgdh1.1*, *ts-pgdh1.2*). Data presented are means ± SD of n = 4. Asterisks indicate significantly different values between EV control plants and *PGDH1*-silenced lines by the Student’s t test (*P < 0.05)

Analysis of the different glucosinolate species revealed no significant differences in the content of the aliphatic glucosinolates (AG) 3-MSOP (3-(methylsulphinyl)propyl), 4-MSOB (4-(methylsulfinyl)butyl), 5-MSOP (5-(methylsulphinyl)pentyl), 4-MTB (4-(methylthio)butyl) and 8-MSOO (8-(methylsulfinyl)octyl) between Mock-treated *PGDH1*-silenced lines and empty vector (EV) control plants (Fig. 4). In contrast, *PGDH1*-silenced lines showed consistent lower levels of the IG I3M (indole-3-ylmethyl) and its processed derivative 1-MO-I3M (1-methoxyindole-3-ylmethyl), while the content of 4-MO-I3M (4-methoxyindole-3-ylmethyl) was not altered (Fig. 4).

Most plant pathogens trigger the production of different phytohormones, which are subsequently part of the innate plant immunity (Brader et al. 2001; Kliebenstein et al. 2002; Mikkelsen et al. 2003; Lorenzo et al. 2004; Dombrecht et al. 2007; Hiruma et al. 2010). Some of these hormone signals also impact the biosynthesis of glucosinolates by inducing the expression of main transcriptional regulators (Frerigmann and Gigolashvili 2014). The content of IGs increases strongly after treatment of plants with jasmonate and this response is mainly mediated by the ATR1/MYB34 transcription factor (Frerigmann and Gigolashvili 2014). Therefore, we further investigated the glucosinolate content in Mock and MeJA treated *PGDH1*-silenced (*ts-pgdh1.1*, *ts-pgdh1.2*) and EV (empty vector) control plants (Fig. 4).

While the content of AGs was not significantly different between *PGDH1*-silenced lines and EV control plants after MeJA treatment, the accumulation of the IG I3M and 1-MO-I3M was significantly reduced (Fig. 4). The content of 4-MO-I3M was slightly, but significantly elevated in *PGDH1*-silenced lines.

In summary, the analysis of glucosinolates in *PGDH1*-silenced lines confirmed that the PPSB is required for the synthesis of IGs in Arabidopsis plants.

### PPSB-mediated serine biosynthesis is required to maintain plant growth at high levels of indolic glucosinolate biosynthesis

**Fig. 5:**
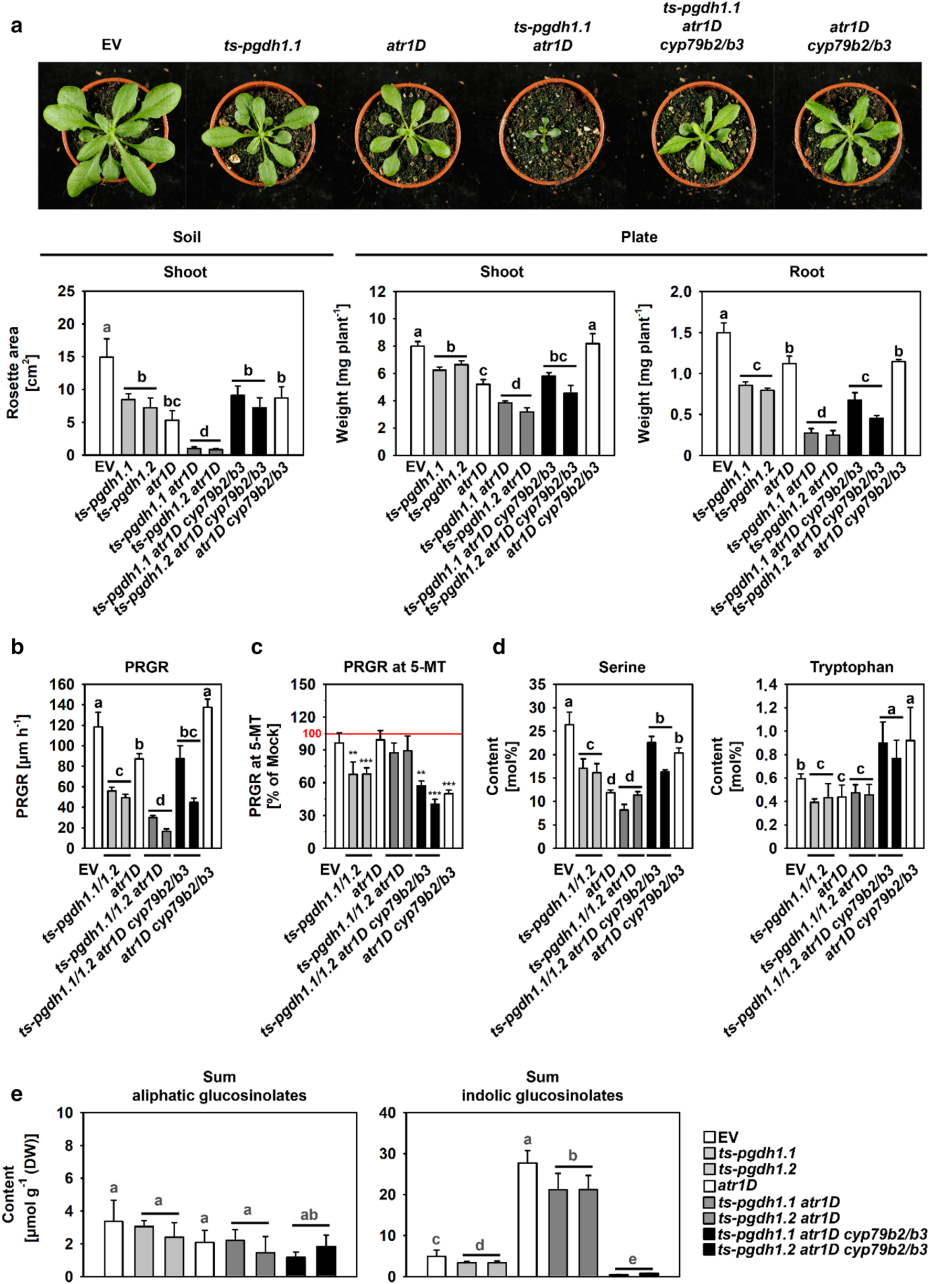
*PGDH1*-deficient plants with enhanced indolic glucosinolate biosynthesis are impaired in growth. **a** Rosette area, shoot and root fresh weight, **b** primary root growth rate (PRGR), **c** relative PRGR of plants grown at 5 µM 5-methyl-tryptophan (5-MT) compared to Mock treated plants in percent, **d** amino acid content in mol percentage, and **e** glucosinolate content of empty vector (EV) control, *PGDH1*-silenced lines (*ts-pgdh1.1 and ts-pgdh1.2*), *atr1D* mutants, *ts-pgdh1.1 atr1D* and *ts-pgdh1.2 atr1D* double mutants, *ts-pgdh1.1 atr1D cyp79b2/b3* and *ts-pgdh1.2 atr1D cyp79b2/b3* quadruple mutants, and *atr1D cyp79b2/b3* triple mutants are shown. Data presented are means ± SE (a), (b) and (c) n > 20; (d) and (e) n = 5. Different letters (a, b, d, e) indicate significantly different values (*P* < 0.05). Asterisks (c) indicate significant different values between Mock treated and 5-MT treated plants by the Student’s t test (*P* < 0.05)

To further study the importance of PPSB-derived serine as precursor for the synthesis of IGs, we generated plants with reduced *PGDH1* expression in the background of *atr1D* mutant plants (*ts-pgdh1.1 atr1D*; *ts-pgdh1.2 atr1D*) (Figs. 5 and S2). These plants mimic pathogen-induced activation of IG biosynthesis by simultaneous limitation of PPSB-derived serine. To ensure that the observed phenotypes originate from enhanced flux into IG biosynthesis, we also generated plants additionally lacking the two cytochrome P450 monooxygenase enzymes CYP79B2 and CYP79B3 (*ts-pgdh1.1 atr1D cyp79b2/b3*; *ts-pgdh1.2 atr1D cyp79b2/b3*). Both enzymes are essential for the conversion of tryptophan into IAOx (indole-3-acetaldoxime), the precursor for the synthesis of IGs, camalexin and (IAA) indole-3-acetic acid (Mikkelsen et al. 2000; Zhao et al. 2002; Bender and Celenza 2009). The genetic background of all mutants was examined in accordance with previous publications (Zhao et al. 2002; Celenza et al. 2005) and only plants with similar expression values for *PGDH1* were selected (Fig. S2).

It has been previously described that reducing the activity of *PGDH1* in Arabidopsis impairs plant development (Benstein et al. 2013). Similarly, previous characterization of *atr1D* mutants also revealed a significant impact of the mutation on plant growth performance (Gigolashvili personal communication). Our study confirms the growth phenotype of these transgenic and mutant plants by showing that the rosette area, the shoot and root fresh weight, and the PRGR (primary root growth rate) were significantly reduced in *ts-pgdh1.1*, *ts-pgdh1.2* lines and *atr1D* mutants (Figs. 5a, b and S2). However, the growth defect of these single mutants was dramatically enhanced in the *ts-pgdh1.1 atr1D* and *ts-pgdh1.2 atr1D* double mutants (Fig. 5a, b). While the rosette area of *ts-pgdh1.1*, *ts-pgdh1.2* and *atr1D* lines was reduced to 60 and 40% of control plants, *ts-pgdh1.1 atr1D* and *ts-pgdh1.2 atr1D* possessed only 10% of the rosette area of control plants. Similarly, the shoot and root fresh weight and the PRGR was substantially lower in *ts-pgdh1.1 atr1D* and *ts-pgdh1.2 atr1D* double mutants compared to the respective single mutants and EV control plants (Fig. 5a, b). Interestingly, all growth parameters (rosette area, shoot and root fresh weight, PRGR) were significantly improved in *ts-pgdh1.1 atr1D cyp79b2/3* and *ts-pgdh1.2 atr1D cyp79b2/3* mutants compared to the *ts-pgdh1.1 atr1D* and *ts-pgdh1.2 atr1D* lines (Fig. 5a, b). Therefore, our data indicated that the enhanced flux of precursors into the IG pathway caused the strong growth phenotype of *ts-pgdh1.1 atr1D* and *ts-pgdh1.2 atr1D* lines.

Plants impaired in tryptophan metabolism have been described to be more sensitive to treatment with the tryptophan analogue 5-methyl-tryptophan (5-MT) (Bender and Celenza 2009). The toxicity of 5-MT is based on its inhibitory effect on the anthranilate synthase enzyme (Miozzari et al. 1977). Inhibition of anthranilate synthase reduces tryptophan biosynthesis and results in tryptophan starvation. Genetic screens for 5-MT resistance in Arabidopsis revealed several mutants altered in the biosynthesis of tryptophan and IGs (Bender and Celenza 2009). While plants with elevated tryptophan and IG biosynthesis, such as the *atr1D* mutant are more resistant to 5-MT, plants with reduced IG biosynthesis are more sensitive (Zhao et al. 2002). The latter results from the capacity of Arabidopsis plants to detoxify 5-MT by sequestration into IGs (Zhao et al. 2002). Growth of *ts-pgdh1.1* and *ts-pgdh1.2* lines at non-toxic (5 µM) 5-MT concentrations reduced the PRGR to 70%, whereas the PRGR of EV control plants was not affected (Fig. 5c). Accordingly, *atr1D* mutants, which are more resistant to 5-MT (Celenza et al. 2005) had no growth benefit at such low 5-MT concentrations. The analysis of *ts-pgdh1.1 atr1D* and *ts-pgdh1.2 atr1D* lines also showed no significant impact of 5-MT treatment on the PRGR. This effect is most likely based on the enhanced sequestration of 5-MT into IGs in these plants, which is supported by our finding that the PRGR of *ts-pgdh1.1 atr1D cyp79b2/3* and *ts-pgdh1.2 atr1D cyp79b2/3* lines grown at 5-MT is reduced to below 60%, and thus on nearly the same level as found for the *atr1D cyp79b2/3* mutant (Fig. 5c).

The impact of the different genetic modifications on the development of these mutants indicated that the metabolism of the amino acid precursor and IGs was substantially altered in these plants. This assumption is supported by changes in the level of serine and tryptophan in the different plant genotypes (Fig. 5d). The mol % of serine was significantly lower in *ts-pgdh1.1*, *ts-pgdh1.2* lines and *atr1D* mutants compared to the EV control plants. This effect was somewhat stronger in *ts-pgdh1.1 atr1D* and *ts-pgdh1.2 atr1D* lines and mitigated in *ts-pgdh1.1 atr1D cyp79b2/3* and *ts-pgdh1.2 atr1D cyp79b2/3* lines. Tryptophan was reduced to a similar level in *ts-pgdh1.1*, *ts-pgdh1.2*, *atr1D*, *ts-pgdh1.1 atr1D* and *ts-pgdh1.2 atr1D* lines, while it accumulated in *ts-pgdh1.1 atr1D cyp79b2/3*, *ts-pgdh1.2 atr1D cyp79b2/3* and *atr1D cyp79b2/3* lines (Fig. 5d). The latter effect is explained by simultaneous stimulation of tryptophan biosynthesis due to the *ATR1D* dominant mutation and blocking of tryptophan conversion into IGs in these plants.

Finally, we analyzed the glucosinolate content in the different genotypes (Fig. 5e). As mentioned before, the contents of all AGs were not altered in *ts-pgdh1.1* and *ts-pgdh1.2* lines, while the contents of IGs were significantly reduced. The *atr1D* mutant revealed no changes in the contents of AGs, but accumulated high contents of IGs (Fig. 5e). Thus, our analysis was in line with previous characterizations of the *atr1D* mutant (Frerigmann and Gigolashvili 2014). The content of IGs also accumulated massively in *ts-pgdh1.1 atr1D* and *ts-pgdh1.2 atr1D* lines, but significantly less compared to the *atr1D* single mutant (Fig. 5e). This finding indicated that *ATR1D*-driven IG biosynthesis represents a dominant factor, although the synthesis of the serine precursor is limited. The analysis of *ts-pgdh1.1 atr1D cyp79b2/3* and *ts-pgdh1.2 atr1D cyp79b2/3* lines confirmed the absence of IGs in plants lacking both CYP79B2 and CYP79B3 enzymes as previously reported (Zhao et al. 2002; Frerigmann and Gigolashvili 2014) and did not reveal changes in the contents of AGs (Fig. 5e).

In summary, induction of IG biosynthesis by simultaneous inhibition of PPSB-mediated serine biosynthesis strongly affected plant growth. This phenotype was mitigated when the flux into the IGs was inhibited. Thus, PPSB-derived serine represents a branch point metabolite required for both, plant growth and IG biosynthesis. Latter was supported by the enhanced sensitivity of *ts-pgdh1.1* and *ts-pgdh1.2* lines against 5-MT.

### PPSB-deficient plants lack growth promotion after infection with the beneficial root endophyte *Colletotrichum tofieldiae*

Previous studies suggested an implication of PPSB and phosphate starvation response of plants, as the transcripts of *PGDH1*, *PGDH2*, as well as *PSAT1* accumulated strongly in Arabidopsis plants cultured in liquid medium in the absence of phosphate (Pant et al. 2015). More intriguingly, this increase in expression seems to be significantly reduced in plants lacking PHR1, the main transcriptional regulator of the phosphate starvation response (Rubio et al. 2001; Pant et al. 2015). We confirmed this potential linkage under more physiological growth conditions by testing the expression level of *PGDH1* within control (EV) and *phr1;phl1* mutant plants, grown for five days at phosphate starvation conditions (Fig. S3). Under these conditions *PGDH1* transcript levels were increased in shoot and root tissue of control plants in response to low phosphate, but not in the *phr1;phl1* mutant background (Fig. S3). Though in relation to transcript regulation of *IPS1* (*INDUCED BY PHOSPHATE STARVATION1*), a well characterized marker of the phosphate starvation response (Franco-Zorrilla et al. 2007), alterations in *PGDH1* expression were rather moderate (Fig. S3).

Previous studies showed that the two transcription factors PHR1 and PHL1 are required for the beneficial interaction between Arabidopsis and *C.t.* under phosphate limiting conditions (Hiruma et al. 2016), suggesting that PHR1/PHL-mediated activation of the PPSB might play a role in this interaction. Analysis of previously published expression data (Hacquard et al. 2016) supported the important role of plant phosphate status in PPSB regulation and showed that *C.t.* infection itself is not sufficient to activate PPSB gene expression (Fig. S4).

**Fig. 6:**
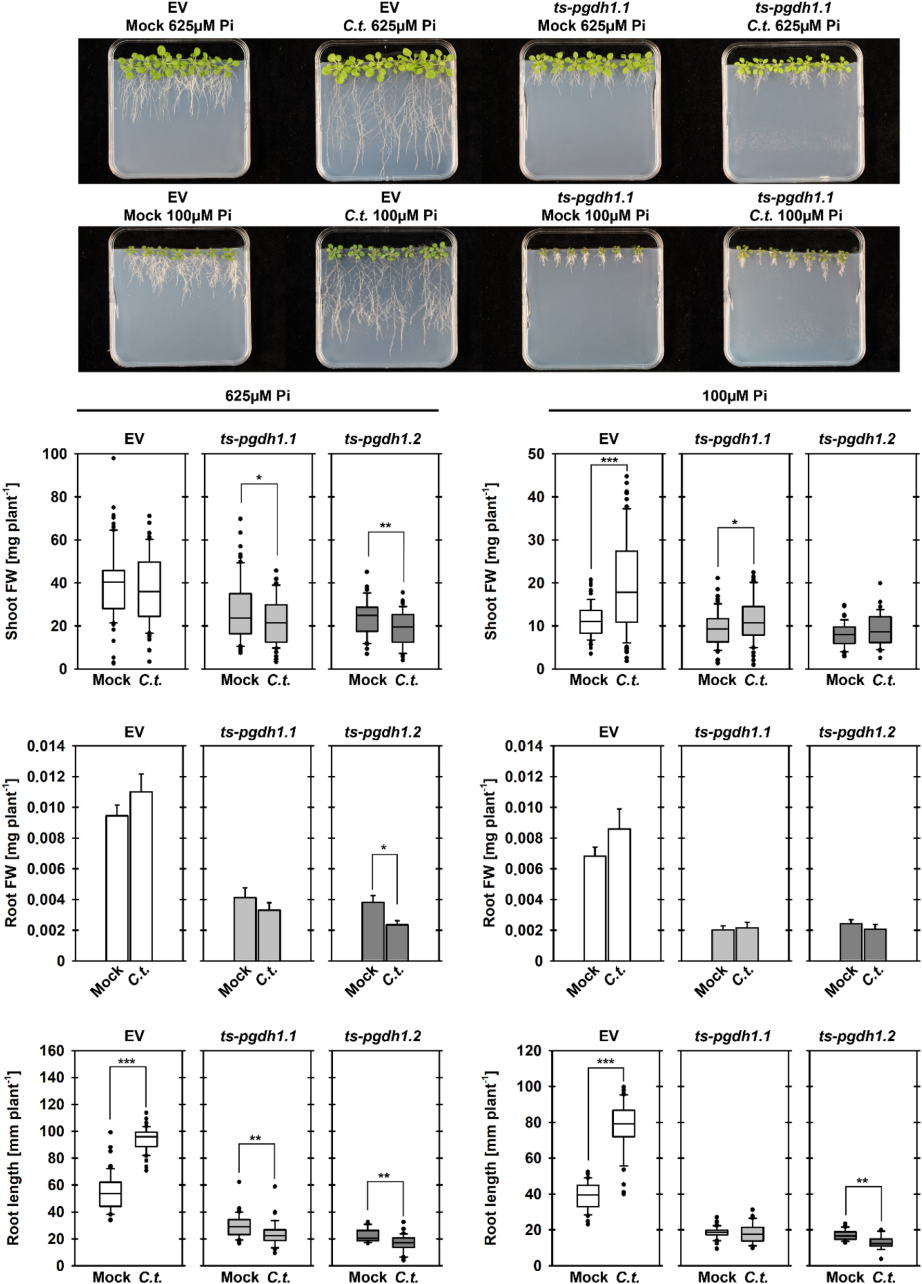
*PGDH1*-deficient plants show specific impairment of *C. tofieldiae*-mediated plant growth promotion. Shoot and root fresh weight, and root length of Mock treated and *C.t.* infected empty vector (EV) control plants and *PGDH1*-silenced lines (*ts-pgdh1.1*, *ts-pgdh1.2*) grown at sufficient phosphate (625 µM Pi) and low phosphate (100 µM Pi) conditions are shown. Data presented are means ± SD of n = 42. Asterisks indicate significantly different values between Mock treated and *C.t.* infected plants by the Student’s t test (*P < 0.05, **P < 0.01, ***P < 0.001)

To gather more information about the role of PPSB-mediated serine biosynthesis in the interaction between Arabidopsis and *C.t.* we infected *ts-pgdh1.1* and *ts-pgdh1.2* lines with *C.t.* and monitored the plant growth response (Fig. 6). Therefore, plants were grown on phosphate sufficient (625 µM) or phosphate limiting (100 µM) conditions and in absence (Mock) or presence (*C.t.*) of the fungus. While shoot fresh weight of EV control plants grown at phosphate sufficient conditions was not altered after infection with *C.t.*, the shoot fresh weight of *PGDH1*-silenced lines was significantly reduced (Fig. 6). The same tendency was also shown for the root fresh weight, whereby the difference was statistically significant only for the *ts-pgdh1.2* line (Fig. 6).

Infection of EV control plants grown at phosphate sufficient conditions increased the length of the primary root by around twofold from 52 to 98 mm, while the root length of *PGDH1*-silenced lines was significantly shorter after *C.t.* infection (Fig. 6).

At phosphate limiting condition *C.t.* infection increased the shoot biomass of EV control plants from around 10 to 17 mg per plant. However, infection of *PGDH1*-silenced lines had no substantial influence on the shoot biomass (Fig. 6). Although the root fresh weight of plants grown at phosphate limiting condition was not significantly altered in both, EV control plants and *PGDH1*-silenced lines, the length of the primary root of EV control plants was significantly longer (Fig. 6). In contrast, the root length of *PGDH1*-silenced lines was not changed (*ts-pgdh1.1*) or even reduced (*ts-pgdh1.2*) after *C.t.* infection (Fig. 6).

To test whether the growth reduction of *PGDH1*-silenced lines after *C.t.* infection was caused by an enhanced susceptibility, we quantified *C.t.* biomass by determining the amount of fungus DNA using qPCR (Frerigmann et al. 2021). As indicated by the qPCR analysis, the biomass of *C.t.* increased in plants grown under phosphate limiting conditions (Fig. S5). However, independent of the phosphate status of the plants, no significant difference in the colonialization with *C.t.* was observed between EV control plants and *PGDH1*-silenced lines (Fig. S5).

Altogether, we confirmed that the PPSB is activated under phosphate limitation and this regulation requires the transcription factor couple PHR1 and PHL1. In addition, we found that *C.t.*-mediated plant growth promotion needs a functional PPSB and growth of *PGDH1*-silenced lines was significantly hampered after *C.t.*-infection, although these plants were not more susceptible against the fungus.

## Discussion

In the last two decades research on the model plant *Arabidopsis thaliana* has progressively increased our understanding of glucosinolate metabolism in plants (Halkier 2016). Most chemical structures and proteins involved in the synthesis, modification and transport of glucosinolates have been identified (Halkier 2016). In addition, the paradigm about the function of glucosinolates in plant defense has been changed by the realization that, in addition to their function in herbivore defense, they also play an essential role in controlling the invasion of various filamentous pathogens (Pastorczyk and Bednarek 2016). Nevertheless, our knowledge on how primary metabolism is adjusted to the requirements of glucosinolate biosynthesis is still fragmentary. The energy cost for the synthesis of glucosinolates in plants is considerable and thus requires a tight cooperation with primary metabolism (Bekaert et al. 2012). Previously, several primary metabolic pathways, such as sulfur assimilation, methionine biosynthesis and the shikimate pathway have been identified as part of a network co-regulated with glucosinolate biosynthesis (Gigolashvili et al. 2007b, 2007a; Hirai et al. 2007; Malitsky et al. 2008; Yatusevich et al. 2010).

In our study, we identified the phosphorylated pathway of serine biosynthesis as a new component of the regulatory network required for the synthesis of IGs in *Arabidopsis thaliana*. Expression analyses revealed that major genes of the PPSB, namely *PGDH1*, *PGDH2* and *PSAT1,* were significantly co-regulated with core genes of the tryptophan biosynthesis pathway (Figs. 1, S1). Tryptophan biosynthesis is up-regulated under high-demand conditions to balance the need for protein biosynthesis with the need for the synthesis of tryptophan-derived secondary metabolites (Niyogi and Fink 1992; Niyogi et al. 1993; Zhao et al. 1998). Several studies showed that this regulation is mediated by the *ATR1*-like clade of transcription factors (Malitsky et al. 2008; Frerigmann and Gigolashvili 2014). When investigating the impact of ATR1/MYB34 transcription factor on the expression of PPSB genes, we found that the transcript levels of *PGDH1* and *PSAT1* were elevated in the *atr1D* dominant mutant (Fig. 2a). Trans-activation of the respective promoter β-glucuronidase fusions by ATR1/MYB34 confirmed this finding (Fig. 2c). Thus, the co-expression of PPSB genes with genes involved in the biosynthesis of tryptophan and IGs is regulated by the same transcription factor.

Interestingly, we found no changes in expression of PPSB genes in loss-of-function *myb-tr* triple mutants, suggesting that the regulation of these genes by *ATR1*-like transcription factors is required only under conditions of a high demand for tryptophan or tryptophan-derived metabolites. The analysis of the amino acid content in *myb-tr* and *atr1D* mutants supports this assumption (Fig. 3a, b). Even though no substantial alterations were observed in *myb-tr* mutants, the contents of some amino acids were significantly changed in *atr1D* mutants (Fig. 3a, b). The most obvious changes were found for serine and asparagine. The content of serine was decreased, whereas the content of asparagine was increased in *atr1D* mutants. While the reduced level of serine in *atr1D* mutants confirmed that serine is required for IG biosynthesis, the elevated level of asparagine indicated increased glucosinolate turnover in these plants (Jeschke et al. 2019). Apart from other functions, asparagine is produced in plants during the hydrolysis of organic cyanides, which are glucosinolate breakdown products (Janowitz et al. 2009). Thus, the increased content of asparagine in combination with the reduced content of serine hinted at an elevated flux through the IG biosynthesis and degradation pathways in *atr1D* mutants. This hypothesis was confirmed by analyzing the amino acid content in *atr1D cyp79b2/b3* triple mutants, which are unable to synthesize IGs (Celenza et al. 2005). Almost all observed changes in the amino acid contents in *atr1D* mutants were reverted in *atr1D cyp79b2/b3* triple mutants (Fig. 3b). Thus, we conclude that the elevated synthesis of tryptophan and subsequently IGs in the *atr1D* mutant induces changes in the amino acid content in these plants and that serine might be a limiting precursor for IG biosynthesis.

When analyzing the contents of glucosinolates in previously characterized *PGDH1*-silenced lines (Benstein et al. 2013), lower levels of IGs were found, whereas the contents of AGs were unaltered (Fig. 4). Jasmonate treatment, which is known to boost particularly the synthesis of IGs (Mikkelsen et al. 2003), caused higher levels of IGs in both, control plants and *PGDH1*-silenced lines (Fig. 4). However, the accumulation of the major IGs I3M and 1-MO-I3M was significantly lower in *PGDH1*-silenced lines compared to the control plants (Fig. 4), providing an additional evidence for a role of the PPSB in IG biosynthesis. Accordingly, *PGDH1*-silenced lines displayed higher susceptibility against inhibition of the PRGR by 5-MT (Fig. 5c), a characteristic trait of plants impaired in IG biosynthesis (Zhao et al. 2002). In summary, our findings provide evidence that serine generated by the PPSB is crucial for the synthesis of IGs in Arabidopsis.

To further investigate the role of the PPSB for IG biosynthesis, we aimed at continuously boosting the metabolic flux into the IG pathway by simultaneously limiting the availability of PPSB-derived serine. Therefore, we generated *atr1D ts-pgdh1* double mutants. We found that the double mutants accumulated high amounts of IGs, nearly similar to the quantities determined in *atr1D* single mutants (Fig. 5e), but their growth was massively impaired (Fig. 5a, b). This finding indicated that the availability of serine for housekeeping functions, such as protein or nucleotide biosynthesis is limiting in these plants. The reduced serine content in the *atr1D ts-pgdh1* double mutants and the reversion of the growth phenotype after the additional blocking of IG formation, achieved by generating *atr1D ts-pgdh1 cyp79b2/b3* quadruple mutants, support this conclusion (Fig. 5a-d). In addition, these data are in agreement with previous findings showing that maintenance of high IG levels takes priority over plant growth in *trp3-1* and *trp2-8* tryptophan-deficient mutants (Müller and Weiler 2000; Brader et al. 2001). Therefore, our data suggest that serine produced by the PPSB represents a branch point metabolite of particular importance for the trade-off between plant growth and IG biosynthesis.

Previous investigations revealed the importance of IG metabolism for controlling the beneficial interaction with the filamentous fungus *C.t.* under phosphate limiting conditions (Hiruma et al. 2016). To prevent transition of *C.t.* from beneficial to pathogenic life-style, Arabidopsis plants control *C.t.* colonization by accumulating glucosinolates under phosphate starvation conditions (Pant et al. 2015; Hiruma et al. 2016). Activation of glucosinolate biosynthesis is triggered in part by *C.t.* infection itself (Hacquard et al. 2016), but also occurs in uninfected plants in response to phosphate deficiency (Pant et al. 2015). In accordance, we found that the expression of PPSB genes was up-regulated in response to phosphate starvation (Fig. 1) and this regulation requires the PHR1/PHL transcription factor couple (Fig. S3). Furthermore, the analysis of publicly available expression data (Hacquard et al. 2016) indicated that *C.t.* infection alone had no influence on the regulation of the PPSB genes (Fig. S4), suggesting that PPSB activation is mainly driven by the phosphate status of the plant.

To further test the significance of the PPSB for this mutualistic interaction, we infected *PGDH1*-silenced plants grown at phosphate sufficient and limiting condition with *C.t.* (Fig. 6). We found that infected *PGDH1*-silenced lines lacked the growth promoting effect observed for control plants independent of the phosphate status (Fig. 6). The shoot biomass of *PGDH1*-silenced lines grown under sufficient phosphate was lower and the length of the primary root was significantly reduced after *C.t.* infection compared to Mock-treated plants. In addition, the gain in shoot and root biomass observed for control plants grown under phosphate starvation conditions after *C.t.* infection was missing in *PGDH1*-silenced lines. However, quantification of fungal growth revealed no significant differences between control plants and *PGDH1*-silenced lines (Fig. S5). Thus, our data indicate that IG-mediated plant immunity in *PGDH1*-silenced lines was sufficient to keep *C.t.* infection in check, but at the cost of plant growth.

## Materials and methods

### Plant growth

In this study all experiments were conducted with *Arabidopsis thaliana* plants of the ecotype Columbia (Col-0). Most of the mutants used in this study have been described previously: *atr1D* single mutant (Bender and Fink 1998); *cyp79b2 cyp79b3* (*cyp79b2/b3*) double and *atr1D cyp79b2/b3* triple mutant (Celenza et al. 2005); *myb-tr* (Frerigmann and Gigolashvili 2014); empty vector (EV) control plants and *PGDH1*-silenced lines (*ts-pgdh1.1* and *ts-pgdh1.2*) (Benstein et al. 2013; Cascales-Minana et al. 2013; Krueger et al. 2017).

The *atr1D ts-pgdh1.1 and atr1D ts-pgdh1.2* double mutants, and the *atr1D ts-pgdh1.1 cyp79b2/b3* and *atr1D ts-pgdh1.2 cyp79b2/b3* quadruple mutants were generated in this study (Fig. S2). Therefore, *atr1D* single mutants and *atr1D cyp79b2/b3* triple mutants were transformed with the *PGDH1*-silencing construct previously used for the generation of the *ts-pgdh1.1* and *ts-pgdh1.2* lines (Krueger et al. 2017). Various double and quadruple mutant lines with an expression level of *PGDH1* similar to *ts-pgdh1.1* and *ts-pgdh1.2* were identified by RT-qPCR and two individual double and quadruple mutant lines were selected for further analysis (Fig. S2). Although the *atr1D* and *atr1D cyp79b2/b3* mutants were previously characterized (Bender and Fink 1998; Celenza et al. 2005), we confirmed the presence of the point mutation in the 5’UTR of the coding region of the *MYB34* gene and the presence of the T-DNA insertion in the *CYP79B2* and *CYP79B3* gene in the generated double and quadruple mutant (for details see Figure legends Fig. S2).

For all experiments, Arabidopsis seeds were surface-sterilized using ethanol (2 × 10 min in 70%, 1 × 10 min 100%) and sown on plates containing half-strength Murashige and Skoog basic salt medium (Duchefa). Seeds were stratified two to four days at 4 °C before the plates were transferred to long-day conditions (16 h photoperiod) for germination. Except where stated otherwise, all experimental plants were grown under controlled conditions (16 h photoperiod 20/18 °C, 150 μmol/m^−2^/s irradiance, 60% humidity) in growth chambers.

### Histological staining

To investigate the influence of MeJA treatment on the activity of the *PGDH1* gene, previously characterized plants expressing the *uidA* (ß-glucuronidase) gene under the control of the *PGDH1* promoter (Benstein et al. 2013) were grown on half-strength Murashige and Skoog medium in the presence (50 µM MeJA) or absence (Mock) of MeJA (Fig. 1b). The GUS signal was visualized by staining seedlings with X-Gluc for 12 h at 37 °C, followed by 3 h destaining of the chlorophyll in 75% ethanol. GUS-stained seedlings were cleared by incubation in Hoyer’s Solution (Anderson 1954) (Mixture of chloral hydrate:water:glycerol 3.0:0.8:0.2), placed on a glass slide and photographed using a Leica S8AP0 binocular microscope and the respective LAS-EZ (2.1.0) software package.

### Expression analysis

Total RNA extraction and RT-qPCR analysis were performed according to Udvardi et al. (2008). The relative quantification of gene expression was conducted using the ∆CT method and the expression values were normalized to *ACTIN*. Except stated otherwise, two technical and five biological replicates were analyzed. For primer sequences see Table S1.

### Trans-activation assay

Cultured Arabidopsis root cells were transformed using the supervirulent Agrobacterium strain LBA4404.pBBR1MCS.virGN54D harboring the constructs for expressing the MYB34 transcription factor (Berger et al. 2007), the anti-silencing protein p19, and the uidA gene under the control of the respective promoters (Fig. 2c). The transformed cells were grown for three to five days and the GUS activity was visualized by adding 100 µL of staining solution (1 mM X-Gluc, 50 mM NaH2PO4, pH 7.1) to 3 ml of the cells and followed by incubation 12 h at 37 °C.

For quantification of the GUS activity, total protein was extracted from 1.5 ml of pelleted root cell culture. The pellet was homogenized and proteins were extracted using 0.5 ml ice-cold extraction buffer (1 mM EDTA, 0.1% v/v Triton X-100, 50 mM NaH2PO4, pH 7.1). After centrifugation for 20 min at 4 °C at 12,000×*g* 25 µl of the supernatant was mixed with 200 µl MUG solution (22 mg 4-methylumbelliferyl-b-d-glucuronide in 50 ml extraction buffer) and incubated at 37 °C. The formation of 4-methylumbelliferone was continuously monitored fluorometrically (excitation, 340 nm; emission, 465 nm) using a plate reader. The linear slope of the curve was determined and the values were normalized to the protein content within the extract. The total amount of protein in the extract was quantified according to the Bradford (1976) method.

### Metabolite analysis

For quantification of amino acids 50 mg of plant material was frozen into liquid nitrogen and homogenized using a ball mill. The amino acids were extracted with 80% ETOH and the extract was diluted 1:10 with water. Twenty microliters of the diluted extract were injected into the HPLC system. Amino acid were separated using a HyperClone 3u ODS (C18)120 150 × 4.6 mm column (Phenomenex) connected to the Dionex UltiMateTM 3000 system (Thermo Fisher Scientific). The HPLC system held a discontinuous flow gradient comprising solvent A (8.8 mM NaPO4, pH 7.5, and 0.2% (v/v) tetrahydrofuran) and increasing proportion of solvent B (18.7 mM NaPO4, pH 7.5, 32.7% (v/v) MeOH and 20.6% (v/v) acetonitrile), at a flow rate of 0.8 ml/min. Quantification was performed by pre-column derivatisation with ortho-phtalaldehyde (OPA; Grace Davison Discovery Science) and subsequently quantified by fluorescence detection of the obtained derivatives (Lindroth and Mopper 1979).

Extraction and quantification of desulpho-glucosinolates was performed as described previously (Gigolashvili et al. 2007b).

### Inhibitor treatment

Seeds were germinated on half-strength Murashige and Skoog medium. Four days after germination seedlings were transferred to vertical plates supplemented with or without 5 µM 5-methyltryptophan (5-MT). The root length of the plants was monitored every day and the primary root growth rate was calculated.

### Fungal infection

Co-cultivation of *C. tofieldiae* and *A. thaliana* was performed as previously described (Frerigmann et al. 2021). Therefore, fungi and plants were co-cultivated on half-strength Murashige and Skoog medium (750 µM MgSO_4_, 625 or 100 µM KH_2_PO_4_, 10.3 mM NH_4_NO_3_, 9.4 mM KNO_3_, 1.5 mM CaCl_2_, 55 nM CoCl_2_, 53 nM CuCl_2_, 50 µM H_3_BO_3_, 2.5 µM KI, 50 µM MnCl_2_, 520 nM Na_2_MoO_4_,15 µM ZnCl_2_,75 µM Fe-EDTA, and 500 µM morpholineethanesulfonic acid-KOH, pH 5.5) with either high Pi (625 µM Pi) or low Pi (100 µM Pi) concentration. The medium was supplemented with 1% nutrient-poor granulated Difco Agar (BD Biosciences). *C. tofieldiae* was pregrown on PDA media at 25 °C for at least one day and fungal suspension (50 mg/ml) was prepared as previously described (Frerigmann et al. 2021). For co-cultivation, stratified sterile Arabidopsis seeds were resuspended in 250 µl sterile water and 20 µl fungal suspension was added. After washing two times with 1 ml of 20 mM MgCl_2_, infected seeds were transferred to high or low Pi plates. For each condition four to five replicate plates with seven seeds per plate were prepared. Plates were placed into a phytochamber and plants were grown at short-day conditions (10 h at 21 °C and 14 h at 19 °C).

## Supplementary Information

Below is the link to the electronic supplementary material.

Supplementary file1 (DOCX 2601 kb)

## Data Availability

The authors confirm that the data supporting the findings of this study are available within the article and its supplementary materials.
