# Supplementary material for: The phosphorylated pathway of serine biosynthesis is crucial for indolic glucosinolate biosynthesis and plant growth promotion conferred by the root endophyte Colletotrichum tofieldiae
Source: Plant Mol Biol. 2021 Aug 23;107(1-2):85–100. doi: 10.1007/s11103-021-01181-5 (PMC8443527; doi:10.1007/s11103-021-01181-5)
Supplement: Supplementary file 1 — Supplementary file1 (DOCX 2601 kb) [file 11103_2021_1181_MOESM1_ESM.docx]

# Supplemental Figures

## Fig. S1


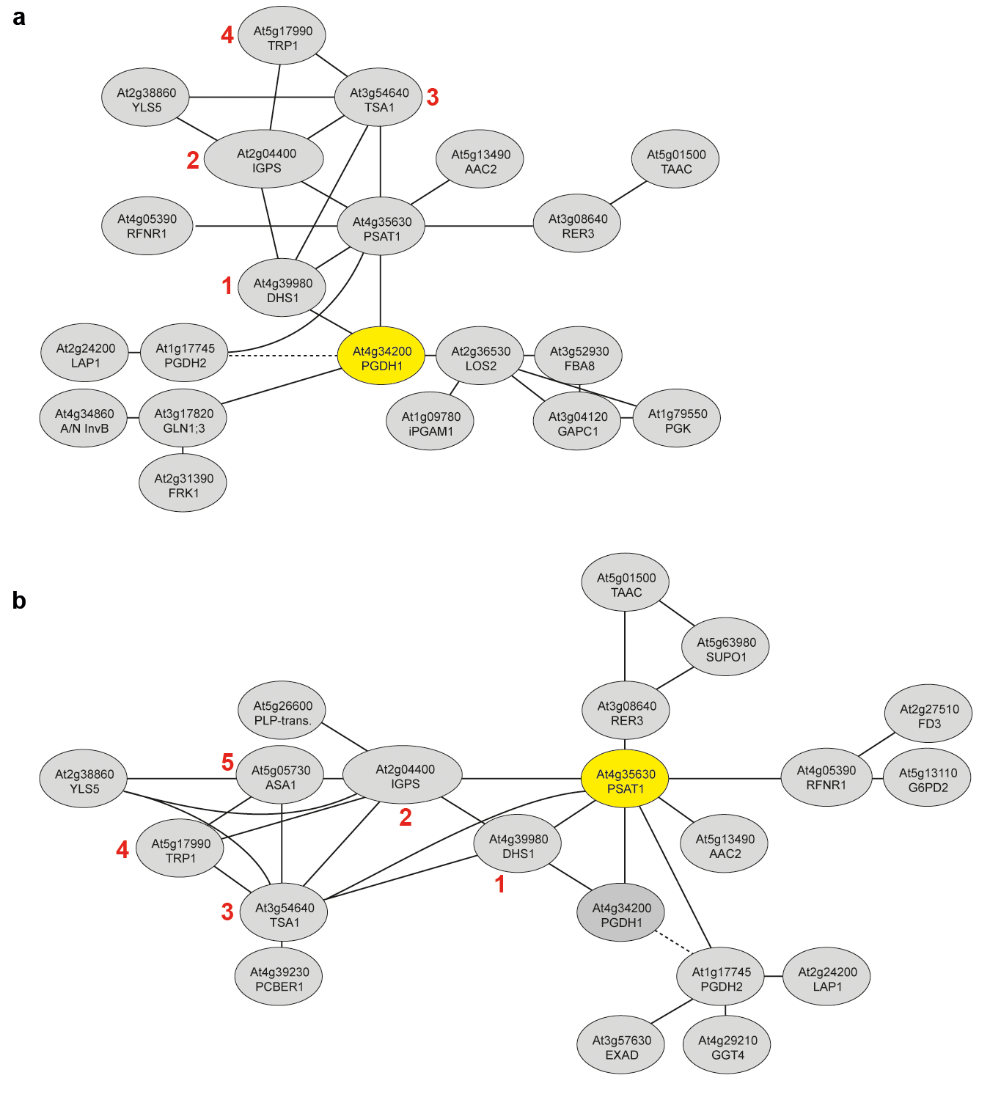


**Fig. S1 Gene correlation network**

Analysis of the gene correlation network of (*a*) *PGDH1* and (*b*) *PSAT1* by using the ATTED Database (<https://atted.jp/>). (1) *DHS* (*3-DEOXY-D-ARABINO-HEPTULOSONATE 7-PHOSPHATE SYNTHASE*); (2) *IGPS (INDOLE-3-GLYCEROL PHOSPHATE SYNTHASE)*; (3) *TSA1 (TRYPTOPHAN SYNTHASE ALPHA SUBUNIT)*; (4) *(PHOSPHORIBOSYLANTHRANILATE TRANSFERASE 1)*; (5) *ASA (ANTHRANILATE SYNTHASE)*

## Fig. S2


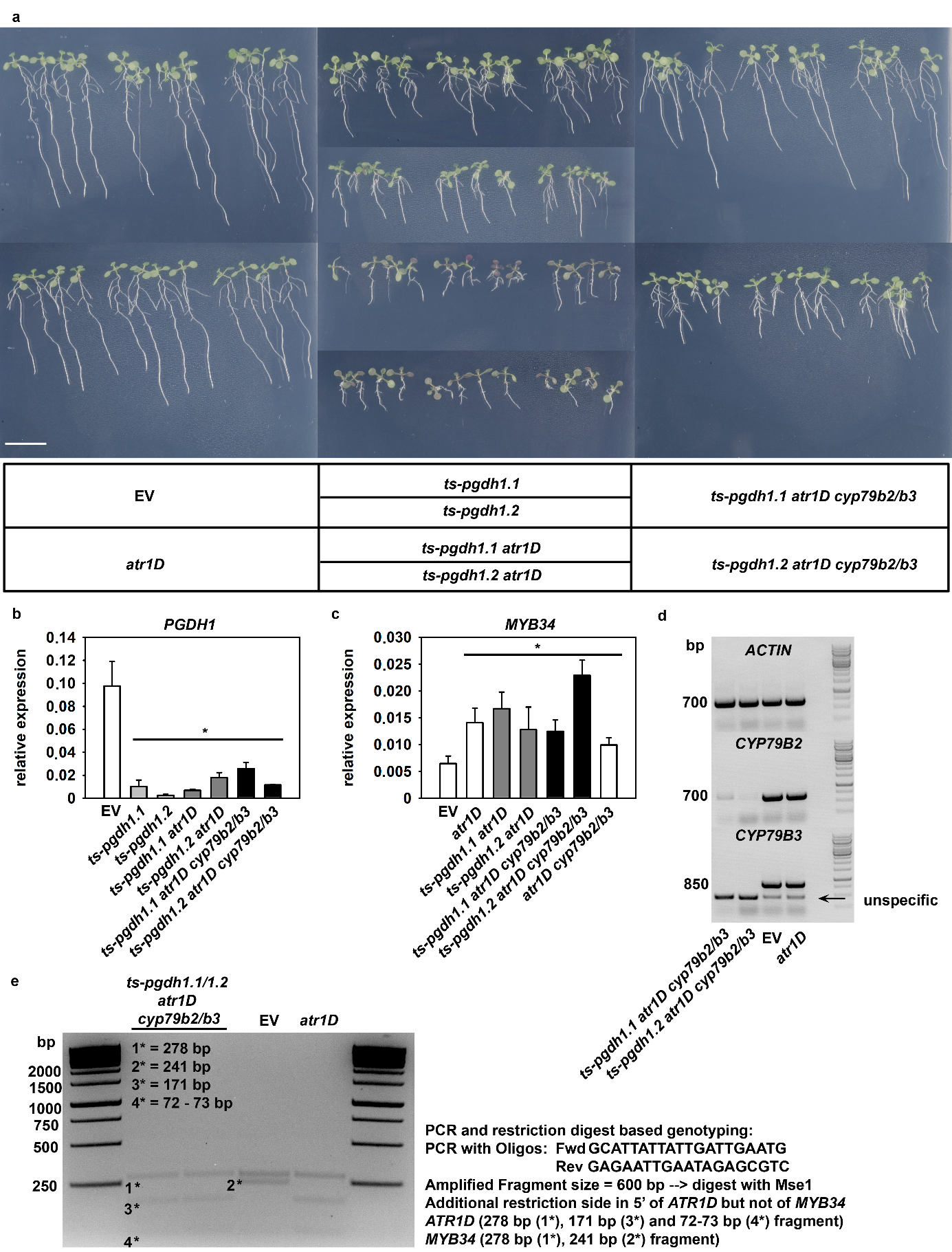


**Fig. S2 Characterization of higher order mutant plants**

*(a)* Root phenotype of empty vector (EV) control plants, *PGDH1*-silenced lines (*ts-pgdh1.1* and *ts-pgdh1.2*), *atr1D* single mutants *atr1d ts-pgdh1.1* and *atr1D ts-pgdh1.2* double mutants, and the *atr1D ts-pgdh1.1 cyp79b2/b3* and *atr1D ts-pgdh1.2 cyp79b2/b3* quadruple mutants. Expression analysis of *(b)* *PGDH1* and *(c)* *MYB34* in the above mentioned plant lines. Results are means ± SD of 5 biological replicates. Asterisks indicate significant differences between EV and the above mentioned plant lines by the Student’s t test (* *P* < 0.05). *(d)* Validation of T-DNA insertion in the *CYP79B2* and *CYP79B2* gene in *atr1D ts-pgdh1.1 cyp79b2/b3* and *atr1D ts-pgdh1.2 cyp79b2/b3* quadruple mutants. *(e)* Validation of point mutation in the 5’UTR of *MYB34* in *atr1D* single mutants and *atr1D ts-pgdh1.1 cyp79b2/b3* and *atr1D ts-pgdh1.2 cyp79b2/b3* quadruple mutants by restriction fragment length polymorphism.

## Fig. S3


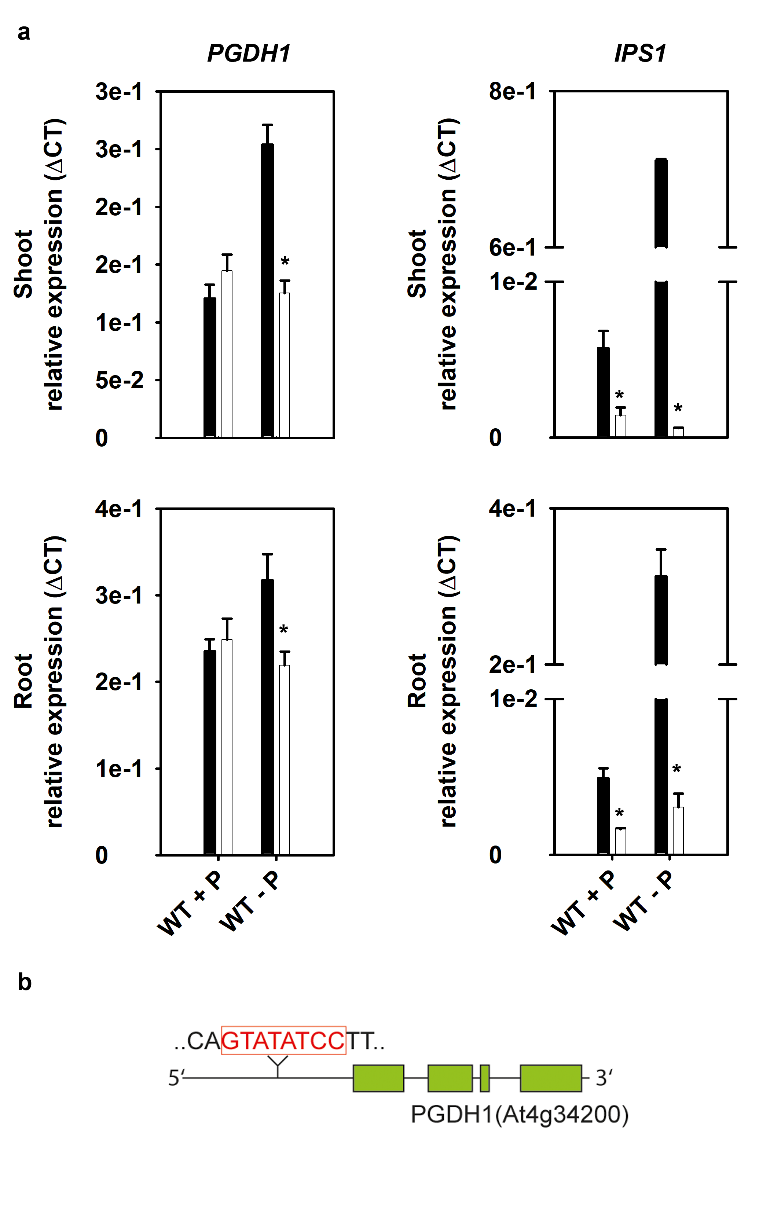


**Fig. S3 Effect of phosphate deprivation on the expression of *PGDH1* and *IPS* genes in wild type and *phr1;phl1* mutant**

Concentrations for high (+P) or low (-P) phosphate conditions were 625 µM Pi and 25 µM Pi, respectively. *(a)* Quantification of transcript levels of *PGDH1* in 13days old EV (black bars) and *phr1;phl1* mutants (white bars) grown at high or low phosphate for 5 days. *SPX1* was analysed as positive control for genetic induction of the phosphate starvation response. Results are means ± SD of 3-7 biological replicates. Asterisks indicate significant differences between EV and *phr1;phl1* mutants by the Student’s t test (* *P* < 0.05). *(b)* Presence of the P1BS element (GNATATNC), the binding site for PHR1, in the promoter regions of the *PGDH1* gene.

## Fig. S4

**Fig. S4 PPSB gene expression data extracted from the RNA-seq dataset published by Hacquard et al., 2016**

Log2 expression fold-changes (logFC) of PPSB genes between *C.t.* infected and Mock-treated plants grown on 650 µM phosphate (plusP, blue) and 50µM phosphate (minusP, red) medium are shown (left graph). Log2 expression fold-changes (logFC) of PPSB genes between 50µM phosphate and 650 µM phosphate in *C.t.* infected (Ct, blue) and Mock-treated (mock, red) plants (right graph). Asterisks indicate significant differences by the Student’s t test (* *P* < 0.05).

## Fig. S5


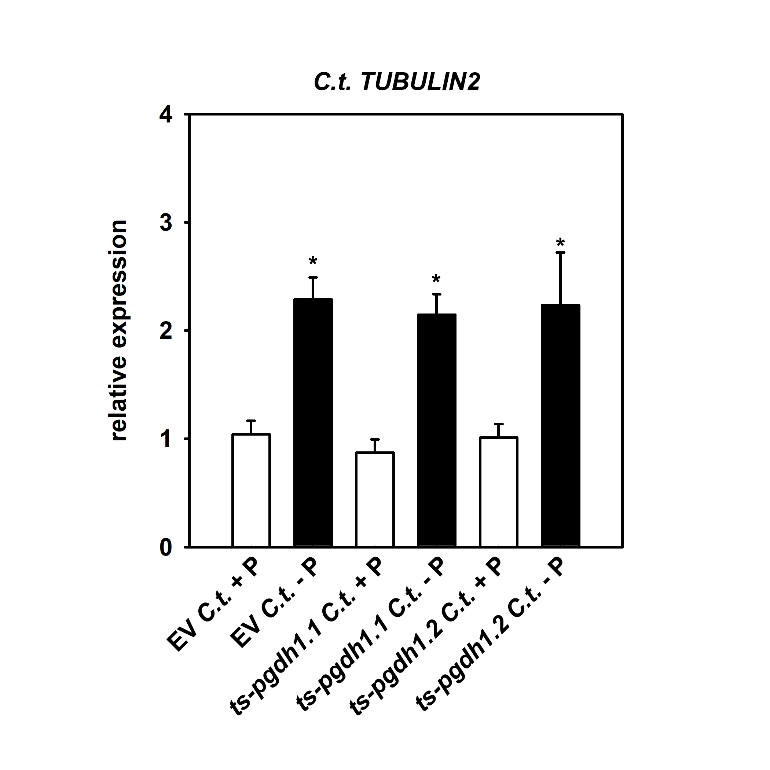


**Fig. S5 Fungal growth is not impaired in *PGDH1*-silenced lines**

Fungal growth in empty vector (EV) control plants and *PGDH1*-silenced lines (*ts-pgdh1.1* and *ts-pgdh1.2*) at high phosphate (+P; 625µM) and low phosphate (-P; 100µM) is shown. Results are means ± SE from three independent experiments containing three or four replicates each (n=9 to 12). Asterisks indicate significantly differences between high and low phosphate by the Student’s t test (* *P* < 0.05).

## Tab. S1

| ***GENE name* (AGI)** | **forward** | **revers** |
| --- | --- | --- |
| **RT-qPCR** |  |  |
| *MYB34 (AT5G60890)* | CACGACTGTCGATAATTTTGGGTTT | CATATTGTCATCTTCGTTCCAGGAA |
| *ASA1 (AT5G05730)* | TGGTGACATGGACATTGCTT | AGATAAGCCACCCACTCACG |
| *TSB (AT5G54810)* | AACAAGCGTTGGAGAAATGG | GATCATCCGGACCTCTGTGT |
| *CYP79B2 (AT4G39950)* | AACAAAAAGAAACCGTATCTGCC | TCCTAACTTCACGCATGCTATCT |
| *PGDH1 (AT4G34200)* | GCCAGTTCCCTGTCTGAATC | CCGGTGAGATTTAAGATTCAAG |
| *PSAT1 (AT4G35630)* | GCTGGTGTCGAAAAGCTTGT | CCGGAAGAAGGAACGAAAACT |
| *ACTIN-1 (AT2G37620)* | CTTGCACCAAGCAGCATGAA | CCGATCCAGACACTGTACTTCCTT |
| *IPS1 (AT3G09922)* | AGACTGCAGAAGGCTGATTCAGA | TTGCCCAATTTCTAGAGGGAGA |
| *C.T. TUBULLIN2* | AGTCTTTCCTGATCCCGACC | AAGTGGCCAGATCAAGTCAA |
| *PP2A (AT1G59830)* | CAAGAGGTTCCACACGAAGGA | TGTAACCAGCACCACGAGGA |
| **PCR** |  |  |
| *gDNA_CYP79B2 (AT4G39950)* | CCGCCGATGAAATCAAACCC | AACTTTTGGGTTACGGCCCA |
| *gDNA_CYP79B3 (AT2G22330)* | AGCCTTTGCTTACCGCTGAT | GTGGAGGATCTCCGGTTTGT |
| *gDNA_ACTIN-I (AT2G37620)* | TGCGACAATGGAACTGGAATG | GGATAGCATGTGGAAGTGCATAC |
| *5’UTR MYB34 (AT5G60890)* | GCATTATTATTGATTGAATG | GAGAATTGAATAGAGCGTC |

**Tab. S1 Primer sequences**
